# Sex differences in blackout: evidence of a relationship between disorders of arousal during sleep and alcohol-related blackout in females

**DOI:** 10.3389/fpsyg.2026.1703614

**Published:** 2026-02-12

**Authors:** Grace M. Elliott, Elena Vidrascu, Madeline M. Robertson, Margaret A. Sheridan, Donita L. Robinson, Charlotte A. Boettiger

**Affiliations:** 1 Department of Psychology & Neuroscience, University of North Carolina at Chapel Hill, Chapel Hill, NC, United States; 2 Bowles Center for Alcohol Studies, University of North Carolina at Chapel Hill, Chapel Hill, NC, United States; 3 Biomedical Research Imaging Center, University of North Carolina at Chapel Hill, Chapel Hill, NC, United States; 4 Department of Psychiatry, University of North Carolina at Chapel Hill, Chapel Hill, NC, United States

**Keywords:** alcohol, binge drinking, blackout, parasomnia, somnambulism

## Abstract

**Rationale:**

Disorders of arousal (DoA) constitute a class of related, heritable conditions that includes sleepwalking, sleep terrors, and confusional arousals. These disorders are defined by behavioral features which are shared with a separate phenomenon, alcohol-related blackout (ARB). Both ARB and DoA are characterized by full or partial amnesia and disinhibited behavior with maintenance of nearly normal motor function. While these disorders share phenomenology, to our knowledge no previous studies have examined the relationship between them. This is an important gap in the literature as the existence of a relationship between DoA and ARB would indicate potential shared underlying pathophysiology and genetic risk, possibly opening new pathways for research.

**Objectives:**

The current study intended to probe the relationship between history of ARB and history of DoA among adults as a first step in determining whether they may be connected by shared neurophysiology, and whether there is a role of sex in that relationship. To control for alcohol intake, we examined whether DoA history interacted with alcohol use to predict ARB likelihood.

**Methods:**

A demographically diverse sample (*n* = 358) of adult (ages 18–72) United States citizens completed an online survey assessing self-reported drinking behaviors, lifetime history of ARB, and lifetime history of DoA episodes via the Munich Parasomnia Screening.

**Results:**

Consistent with the literature, greater levels of past year alcohol misuse predicted higher likelihood of ARB in our sample. A novel finding is that history of DoA episodes also interacted with alcohol use to predict higher likelihood of ARB in females only.

**Conclusion:**

Female individuals with a history of DoA may be more susceptible to experiencing ARB than individuals without such a history, suggesting that the two states may share a neurophysiological foundation.

## 1 Introduction

Alcohol-related blackout (ARB) is a physiological state defined by full or partial anterograde amnesia after drinking, with maintenance of mostly intact motor function and the capability to engage in complex tasks (Lee et al., 2009; White, 2003). Although it is by definition induced by drinking, ARB does not appear to just be an extreme form of intoxication, but rather a distinct state only experienced by some who engage in risky drinking (Wetherill and Fromme, 2016). ARB episodes have been associated with both increased risk of injury during intoxication (Mundt et al., 2012) and increased risk of experiencing sexual assault (Wilhite et al., 2018) in college students. In a twin study, individuals with a history of blackout were at an increased risk of later death due to any cause compared to their identical counterparts without ARB history (Sipilä et al., 2016). Experiencing an ARB was associated with increased odds of reporting a number of other alcohol-related harms in a sample of young adults, including academic and relational problems (Hingson et al., 2016). Even beyond the risks associated with binge drinking, ARBs present risk of serious injury or even death, which makes understanding patterns in their incidence a pressing public health issue.

While ARB is strongly associated with certain behaviors (e.g., binge drinking, drinking rapidly, consuming liquor), these behaviors are not fully predictive of an episode (Jennison and Johnson, 1994; Labrie et al., 2011; Merrill et al., 2019; Wechsler, 1994; White, 2003). A blackout episode can be defined as one of two types: partial (fragmentary) or total (en bloc). A partial blackout is one in which some memory of events is maintained, and details can be recalled when prompted (Goodwin et al., 1969). A total blackout is one in which an entire period is completely missing from an individual’s memory (Goodwin et al., 1969). Partial blackouts are reported more frequently and by a higher number of individuals than are total blackouts (Rose and Grant, 2010; Wetherill and Fromme, 2016). Among individuals who report drinking alcohol, between 30 and 50% will ever experience a blackout (Rose and Grant, 2010; Chartier et al., 2011; Hallet et al., 2013). Among individuals with a clinical diagnosis of an Alcohol Use Disorder (AUD), a large (*n* = 3,292) epidemiological study reported correlations ranging from *r* = 0.35 to 0.43 between lifetime AUD diagnosis and lifetime history of blackout (Davis et al., 2019; Goodwin et al., 1969). This discordance suggests that there are biological factors that predispose some individuals to entering the blackout state—an assumption supported by evidence that susceptibility to experiencing blackouts is heritable and related to family history of problem drinking (Davis et al., 2019; Jennison and Johnson, 1994; Nelson et al., 2004; Wetherill and Fromme, 2016). While excessive alcohol consumption is required to trigger an ARB, it is not sufficient, suggesting that ARBs result from individual and environmental factors interacting with alcohol use.

Disorders of arousal (DoA) are certain parasomnias– sleepwalking, sleep terrors, and confusional arousals—that involve episodes of wakefulness during non-rapid eye movement (NREM) sleep (American Academy of Sleep, 2005). Similar to ARB, DoA are defined by periods of intact motor function with behavioral disinhibition and partial or full amnesia of events (Baldini et al., 2019). The DoA differ from ARB in the circumstances under which they can be observed; DoA episodes happen during sleep, while ARB are triggered by acute alcohol intoxication. The literature indicates that both DoA and ARB susceptibility are heritable (Davis et al., 2019; Hublin et al., 1997; Kales et al., 1980; Lecendreux et al., 2003; Nelson et al., 2004), and both are associated with genetic and physiological factors relating to GABAergic inhibition of brain activity (Dick et al., 2006; Oliviero et al., 2007). We hypothesized that DoA and ARB may share latent predisposing factors, as they are strikingly similar in their presentation. Both represent episodes of breakthrough motor activity in states of widespread neural suppression. Our lab previously found evidence of shared neurophysiological markers between the two states, with reduced resting-state EEG alpha power in the sensorimotor cortex being associated with ARB history in females in one sample and correlated with DoA severity in another sample of young adults (Elliott et al., in press). If ARB and DoA share an underlying pattern of irregular brain function, with episodes triggered by alcohol intoxication or NREM sleep, we would expect to see overlap in the populations who experience ARB and DoA. We are particularly interested in these states as reflections of atypical cortical inhibition allowing for nonuniform suppression of function (motor function vs. memory storage). If this hypothesis was supported, it would allow for the use of the DoA literature as a theoretical scaffolding for our understanding of ARB. The mechanisms of ARB are inherently difficult to study in humans retrospectively due to the associated amnesia, and difficult to study directly due to ethical and logistical concerns. Establishing a connection between these states would provide direction for future work on ARB.

The extant literature on blackout is equivocal on whether there are sex differences in risk, although several studies suggest that females may be more vulnerable (Marino and Fromme, 2015; Richards et al., 2023). Given that females tend to have lower body weights and higher body fat percentages than males, it is difficult to distinguish the role of neurophysiology from pharmacokinetics in this vulnerability (Bredella, 2017). There is evidence that the effects of alcohol on brain structure and function can differ according to sex, both in acute and in chronic use (Verplaetse et al., 2021). With regard to the DoA, childhood-onset sleepwalking is more common in females than in males, and heritability of adult-onset sleepwalking seems to differ according to sex (Hublin et al., 1997). In a sample of adults seeking treatment for sleepwalking, women were more likely to report experiencing more than two episodes of sleepwalking per week and more likely to report comorbid sleep terrors (Labelle et al., 2013). A recent video analysis demonstrated differences in behaviors performed during sleepwalking episodes between males and females (Correa et al., 2024). Interestingly, in insomnia patients prescribed zolpidem, sleepwalking as a side effect was more common in females (Banjeree and Nisbet, 2011; Hwang et al., 2010). Given that zolpidem, like ethanol, increases activity at GABA_A_ receptors, this suggests that the role played by this class of drug in amnestic episodes may be sex-dependent (Crestani et al., 2000; Most et al., 2014). Documentation of sex differences in DoA triggers and manifestations and sex differences in the interplay between alcohol and the brain suggests that there may be important qualitative differences in how these states arise in males and females. Additionally, sex differences in GABA-modulating steroids also suggest that any link between DoA and ARB may depend on sex, as GABAergic function is implicated in both (Oliviero et al., 2007; Wirth et al., 2007; Rose and Grant, 2010).

To test the hypothesis that ARB and DoA may share predisposing factors, we designed and disseminated an online survey to assess participants’ alcohol use history, ARB experiences, and sleep history. The primary goal of this study was to determine whether there is a statistically significant relationship between DoA and ARB in a random sample of American adults, even when controlling for other variables known to contribute to blackout risk, particularly frequency and rate of drinking. We predicted that DoA history would interact with alcohol use such that DoA history increases ARB likelihood more steeply as drinking increases. We also predicted that this relationship may differ according to sex. We hypothesized that the existence of DoA history at any point in one’s life reflects inherent neurophysiological factors that increase susceptibility to ARB in the context of hazardous alcohol use, which is why we chose to use a lifetime DoA variable in these analyses.

As mentioned previously, those who experience ARBs are more susceptible to experiencing alcohol-related injuries and consequences than are other drinkers (Hingson et al., 2016; Sipilä et al., 2016; Studer et al., 2019). In this project, we aimed to determine whether DoA history was associated with blackout risk in adults, as a first step in identifying potential markers of ARB predisposition. Ultimately, we hope that this vein of research could help mitigate the public health burden of ARBs by identifying those at high risk of experiencing them.

## 2 Materials and methods

### 2.1 Participants and procedure

An online survey battery created via the secure data capture application, Research Electronic Data Capture (REDCap) (Harris et al., 2009), was distributed to participants via Prolific,^1^ an online data platform that allows for the recruitment of a nationally representative sample (Palan and Schitter, 2018). Prior to data collection we established recruitment targets for age, sex, race, and ethnicity to ensure that our sample would be broadly representative of the adult population in the United States. Our only eligibility requirements were age 18+ years, location in the United States, and fluency in English—which allowed for the collection of a more diverse sample than is often seen in psychological research. Initially, *n* = 575 responses were recorded, with *n* = 14 failures to complete the e-consent form and *n* = 13 duplicate submissions. In order to confirm that participants read and understood the survey questions, a series of attention checks were randomly inserted throughout the survey battery. Only participants with accuracy of 80% or higher on the attention checks were included in the final data set used for analysis. Of *n* = 548 participants who completed our survey, *n* = 30 were excluded on this basis. Dataset accuracy was manually checked by the research team, and payments were distributed to participants through Prolific. Participants were paid $3.70 to complete the online survey. All study procedures were approved by the Office for Human Research Ethics at the University of North Carolina at Chapel Hill.

### 2.2 Measures

Data collection for this study comprised a one-time online self-report survey battery, which included an online consent form and multiple questionnaires. The questionnaires included in the analyses reported here were the Alcohol-Related Blackout Questionnaire (ARBQ; see Supplementary material), the Carolina Alcohol Use Pattern Questionnaire (CAUPQ) (Elton et al., 2021), the Munich Parasomnia Screening (MUPS) (Fulda et al., 2008), the Family Tree Questionnaire (FTQ) (Mann et al., 1985), the Adult Self-Report (ASR) (Achenbach and Rescorla, 2003), an abbreviated version of the Drug Use Screening Inventory (DUSI) (Skinner, 1982), and questions assessing demographics and socioeconomic status. Due to the potentially sensitive nature of questions regarding alcohol and substance use, participants were not required to respond to all questions in order to complete the survey and to receive payment.

#### 2.2.1 Alcohol-related blackout questionnaire

The ARBQ is a series of questions we chose to capture lifetime history of blackouts resulting from alcohol use. This survey distinguishes between total and partial blackouts and allows for temporal specification of blackout frequency across three time periods: before age 18, between ages 18 and 21, and during the past year. The full ARBQ used here is available in the Supplementary material. Although this measure has not been validated, Cronbach’s *α* for the 20 ordinal response items included on the ARBQ was 0.940 in this sample. Past year blackout score was strongly correlated with past year binge drinking score (*r* = 0.657, *p* < 0.001) and with Alcohol Use Disorder Identification Test (AUDIT) score (*r* = 0.594, *p* < 0.001), providing justification for the use of these questions in the following analyses. For the current study, responses assessing number of partial and total blackouts during the past year were used to create a binary past year blackout variable. Any individuals who endorsed 0 partial blackouts and 0 total blackouts during the past year were coded as 0, and any individuals who endorsed 1 + partial or total blackouts during the past year were coded as 1. This binary variable was used as the dependent variable in the following binary logistic regression analyses. Similarly, responses assessing history of blackouts were used to create lifetime and past year continuous blackout variables, which were used to determine bivariate correlation values between variables. Responses on each of the five ARBQ questions corresponding to lifetime blackout history (see Supplementary material) were averaged to create the lifetime variable, and the past year responses were averaged the same way.

We chose to use past year blackout as the dependent variable in our models because the alcohol use measures collected (CAUPQ and AUDIT) specifically assessed drinking over this period. Given the causal relationship between alcohol use and ARB, we had to gather data about drinking frequency, rate, and volume to model ARB likelihood over a specific time period. Additionally, given the large age range of this sample, we wanted to standardize the reporting period such that we were not asking some individuals to recall drinking and blackouts over a 50-year span and others over a 2-year span, for example. As drinking behavior is strongly predictive of ARB, we felt it was critical to limit the outcome (ARB likelihood) to a time period during which we had sufficient data regarding alcohol consumption, and that was equally recent for all participants regardless of age.

#### 2.2.2 Carolina alcohol use pattern questionnaire

The CAUPQ assesses typical drinking behaviors during multiple periods of the lifespan: under the age of 18, between 18 and 21, and during the past year. Items collected include frequency of intoxication, quantity typically consumed, and percentage of drinking episodes approached with the intention of becoming drunk. Ordinal scale responses to the question “Before the age 18, how often did you have 5 or more drinks (4 or more if you are female) containing any kind of alcohol within a two-hour period?” were transformed according to the method described in (Elton, Faulkner, et al., 2021). Specifically, responses of “never” were coded as 0, responses of “1–3 times” were coded as 2, responses of “4–6 times” were coded as 5, and responses of “7–12 times” were coded as 9.5. Responses of “2-3 times per month” were coded as 30 (2.5 times 12) and then multiplied by (18-reported age of first binge), responses of “weekly” were coded as 52 and then multiplied by (18-reported age of first binge), and responses of “> once/week” were coded as 104 (2 times 52) and then multiplied by (18-reported age of first binge). This transformed value was used to approximate total number of binges before age 18 and was included as a covariate in the regression analyses.

We quantified past year alcohol misuse using past year “binge score” (Townshend and Duka, 2002). This metric is derived from number of episodes of drunkenness during the past 6 months, percentage of drinking days approached with the intention of becoming drunk, and typical rate of drinking during the past 12 months (Townshend and Duka, 2002; Elton et al., 2021). Crohnbach’s alpha (standardized) for the three items included in the binge score in this sample was 0.609, and binge score was highly correlated with total AUDIT score (*r* = 0.676, *p* < 0.001), providing evidence for its validity as measure of recent alcohol use. Alcohol misuse and binge score are used interchangably here.

#### 2.2.3 Munich parasomnia screening

The MUPS is an assessment of multiple parasomnias, including the DoA. Responses on the MUPS concerning the three parasomnias classified as DoA were used to create a binary variable representing history of DoA episodes. Any participant who endorsed having experienced sleepwalking, confusional arousals, or sleep terrors during their lifetime was coded as 1, and those with no history were coded as 0. This binary variable was used as a factor in our models.

We also created an ordinal variable representing DoA severity to be used in bivariate correlation analyses. For each of the three questions corresponding to a DoA episode type, a response of “Never observed” was coded as 0, a response of “Observed years ago” was coded as 1, and a response of “Currently observed” was coded as 2. Responses were summed to create a variable with values between 0 and 6, referred to here as DoA severity. The MUPS has previously been validated (Fulda et al., 2008), and Cronbach’s alpha for this measure in our sample was 0.839.

#### 2.2.4 Family tree questionnaire

The FTQ is a self-reported measure of family history of problem drinking, the primary outcome variable of which is the density of first-degree relatives whom the individual recalls displaying problematic drinking behaviors. The family tree density variable included as a covariate in our models was calculated by summing the number of relatives reported to have problems with alcohol and dividing it by the total number of first-degree relatives reported for each participant. We included this variable as a control in our analyses because family history of AUD has previously been associated with blackout susceptibility (Marino and Fromme, 2015).

#### 2.2.5 Adult self-report

The ASR is a 126-item scale assessing multiple aspects of psychopathological thoughts and behaviors in adults, including symptoms of depression, anxiety, and ADHD. After the exclusion of certain questions pertaining to sensitive information, the ASR administered here included 124 items. For each subscale, mean imputation was used to calculate scores for any participants missing less than 20% of responses for that scale. These mean imputed values were used to calculate total psychopathology scores for each individual who answered 80% or more of the items on each subscale. This total score was used as a covariate in our models. The ASR has been validated repeatedly (Achenbach and Rescorla, 2003; Ivanova et al., 2015), and Cronbach’s alpha based on standardized items for this measure in our sample was 0.967.

#### 2.2.6 Drug use screening inventory

We used a 10-item substance use scale adapted from the DUSI assessing the frequency of use for 10 different substances over the past 6 months (from no use up to daily use). Given that one item assesses alcohol specifically, we calculated a total substance use score excluding this item to avoid unnecessary collinearity with binge score in our models. Total substance use was included as a covariate in our models to control for recent use of substances other than alcohol that can cause or contribute to memory loss. Cronbach’s alpha (standardized) for the items included in our total substance use score was 0.604.

#### 2.2.7 Demographic and socioeconomic measures

We collected data on a number of demographic measures, including sex, gender, age, and race, as well as socioeconomic measures, including education level. Race was binarized as white or non-white racial identity, and this binary variable was included as a covariate in our analyses to control for the role of stresses associated with minority group identification in drinking behavior and consequences (Harris et al., 2022; Pittman et al., 2019). Age was included as a covariate in all analyses, and models were run independently for males and females. Maximum education attained was collected according to an ordinal scale from 1 to 20, with 1 indicating completion of first grade and 20 indicating education beyond a Master’s degree, including the completion of a PhD or professional degree. This ordinal variable was included as a covariate in our models.

### 2.3 Data analyses

The total sample collected in this dataset included 518 individuals. Of these, 497 individuals had complete datasets with regard to the variables of interest for this paper. One individual lacked sufficient data from the ASR, and 20 were missing data required to calculate FTQ density or binge score (alcohol misuse score). To isolate biological factors in our sex-specific analyses, we also excluded 27 individuals who reported a gender identity incongruent with their sex at birth. Finally, we excluded 112 participants who reported not consuming any alcoholic drinks in the past year according to the CAUPQ (item 4) or the AUDIT (item 1), as ARB would not be possible for these individuals. The remaining 358 individuals were included in the analyses described below. Little’s MCAR analysis did not indicate the existence of any pattern in these excluded individuals with respect to the variables included in the model (*p* = 0.279). All analyses were performed in SPSS version 29.0.1.0.

We first ran bivariate correlations to determine whether any relationships existed between the variables of interest in this study. This initial analysis did indicate that there exists a relationship between ARB susceptibility and DoA severity, but it did not control for other factors that contribute to risk of ARB; many of which have been well documented, including degree of alcohol use (Labrie et al., 2011). As we were interested in whether DoA history increased blackout susceptibility, we used a binary logistic regression model with binary past year blackout history as the dependent variable. Thus, we next wanted to determine whether this relationship between DoA history and ARB remained when we controlled for these factors, especially given that those with a history of DoA and those without differed significantly in some variables relevant to blackout history (see Table 1). We chose to use binary logistic regression to model the relationship between DoA history and ARB vulnerability in males and females separately. We made this choice because sex differences in how alcohol affects the brain have been repeatedly documented (Pfefferbaum et al., 2001; Van Doorn et al., 2025), and there is evidence for sex differences in DoA manifestation (Correa et al., 2024). We suspected that if any relationship did exist between DoA and ARB, it may differ qualitatively in males and females. The dependent variable in these models was past year blackout history, rather than lifetime history, because we wanted to have temporal consistency between our alcohol use measure (past year binge score) and our dependent variable. These models were intended to assess whether history of DoA contributed to blackout risk over a limited period of time, while accounting for alcohol use during that period.

**Table 1:** Mean (±SD) values for variables in the model by sex and DoA group, with results of independent samples *t*-tests comparing between sexes and between DoA groups within sexes.

Variable	Full sample (*N* = 358)	Males—no DoA (*n* = 121)	Males—DoA (*n* = 47)	Females—no DoA (*n* = 111)	Females—DoA (*n* = 79)
Binge score	13.18 (±17.25)	11.27 (±11.59)	19.13 (±29.71)	11.02 (±10.67)	15.60 (±20.87)
Number of past year blackouts	1.20 (±3.36)	1.14 (±3.26)	1.96 (±5.88)	0.55 (±1.45)**	1.75 (±3.26)**
Number of binges before 18	15.01 (±65.99)	12.46 (± 43.20)	24.82 (± 83.64)	9.82 (± 38.88)	20.37 (± 103.29)
Total ASR score	45.59 (±34.75)***	30.96 (±26.13)***	53.10 (±29.77)***	39.56 (±28.10)***	71.99 (±41.41)***
Family history density	0.17 (±0.18)	0.14 (±0.18)	0.19 (±0.18)	0.14 (±0.16)***	0.24 (±0.20)***
Age	36.19 (±13.63)	38.70 (±13.78)*	33.30 (±11.39)*	35.41 (±13.47)	34.58 (±14.36)
Substance use score	12.94 (±5.28)	12.19 (±4.45)*	14.64 (±5.74)*	11.96 (±4.23)**	14.46 (±6.78)**
Education level	15.20 (±2.16)	15.53 (±2.02)	15.11 (±2.07)	15.19 (±2.21)	14.77 (±2.29)

***p < 0.001, **p < 0.01, *p < 0.05.

Binary logistic regressions were performed in SPSS version 29.0.1.0 using the regression function. We ran the model separately in males (*n* = 168) and females (*n* = 190). For both models, we included age, education level, family history density of problem drinking, number of binge episodes before the age of 18, substance use score, psychopathology according to total ASR score, and past year binge score as covariates. Non-white racial identity and binary DoA history were used as factors. We chose to include a binary white/non-white race variable as previous research has indicated that non-white racial identity is associated with increased risk of certain alcohol-related consequences including higher blackout frequency, possibly relating to the stress induced by experiencing discrimination (Harris et al., 2022; Pittman et al., 2019). Family history of problem drinking, which was collected as a percentage of relatives with a history of hazardous alcohol use, was transformed to a *z*-score before inclusion in the model, as the raw data were overdispersed and right-skewed.

We ran both models including only the variables of interest, and then ran them again with an interaction term between DoA history and binge score included to test whether the relationship between alcohol use and likelihood of reporting a blackout was different for those with a history of DoA and those without. There is a well-established and strong relationship between heavy alcohol use and blackout likelihood (White, 2003). This relationship is causal, which means that we did not anticipate that DoA would independently influence ARB risk in the absence of alcohol use, but that it would moderate the relationship between alcohol use and ARB likelihood such that the slope of the increase in ARB probability with increasing binge score would be steeper in those with a history of DoA. We also ran a restricted version of the models, including only binge score, age, DoA history, and the DoA history by binge score interaction as independent variables. This was done because our full models included a high number of predictors relative to the low number of outcome events and small sample of DoA positive participants. The results of the restricted model were used to validate the results of the full model.

## 3 Results

### 3.1 Sample characteristics

The demographic breakdowns for the full sample are displayed in Table 2. We recruited this sample to be roughly representative of the U. S. adult population with regard to sex, race, and ethnicity. With regard to sex and gender, 46.9% of participants were male and 53.1% were female. In terms of race, 73.7% of participants were White, 15.6% were Black, 8.7% were Asian, Native Hawaiian, or Pacific Islander, and 1.1% were Native American or Native Alaskan. In terms of ethnicity, 20.9% of participants identified as Hispanic. Participant ages ranged from 18 to 72 years old (mean 36.2 years ± 13.6). With regard to education, 43.0% of this sample reported holding a Bachelor’s degree, and 15.1% reported holding a Master’s degree or higher. Much of what we know about the prevalence of and risk factors for ARB has come from research conducted with samples of undergraduates, which means that this dataset is uniquely inclusive in terms of age and education level. Demographic variables split according to sex and blackout history are available in Table 3 (split by past year blackout history) and in Supplementary Table S1 (split by lifetime blackout history). Finally, 23.8% of this sample reported a total Alcohol Use Disorder Identification Test (AUDIT) score of 8 or greater, indicating hazardous alcohol use (Saunders et al., 1993).

**Table 2:** Sample demographic information.

Factor	*N* or mean	% of total sample or SD
Sex/gender		
Male	168	46.9
Female	190	53.1
Race		
White	264	73.7
Black	56	15.6
Asian/Native Hawaiian/Pacific Islander	31	8.7
Native American/Native Alaskan	4	1.1
Prefer not to answer	9	2.5
Ethnicity		
Hispanic	75	20.9
Non-Hispanic	283	79.1
Age (mean = 36.19, SD = 13.63)		
18–27	127	35.5
28–44	129	36.0
45+	102	28.5
Education level		
Did not complete high school	4	1.1
High school diploma or equivalent	89	24.9
Associate’s degree	57	15.9
Bachelor’s degree	154	43.0
Master’s degree	43	12.0
PhD or professional degree	11	3.1
Typical drinks per drinking day	3.30	± 3.20
Typical drinking rate (drinks per hour)	1.37	± 1.16
AUDIT total score	5.47	± 5.56

Values for the categories in “Race” exceed the total sample size, as some individuals endorsed identifying with more than one race.

**Table 3:** Demographic variables by sex and past year blackout groups (total and/or partial blackout).

Factor	Full sample (*N* = 358)		Males—no AIB (*n* = 124)		Males—AIB (*n* = 44)		Females—no AIB (*n* = 131)		Females—AIB (*n* = 59)	
	*n*	%	*n*	%	*n*	%	*n*	%	*n*	%
Race										
White	264	73.7	89	71.8	35	79.5	96	73.3	44	74.6
Non-White	94	26.3	35	28.2	9	20.5	35	26.7	15	25.4
Ethnicity										
Hispanic	75	20.9	26	21.0	9	20.5	24	18.3	16	27.1
Non-Hispanic	283	79.1	98	79.0	35	79.5	107	81.7	43	72.9
Education level										
Bachelor’s Degree or higher	208	58.1	74	59.7	31	70.5	73	55.7	30	50.8

### 3.2 Alcohol-related blackout findings

Of the 358 participants included in our analyses, 31.8% reported having experienced a total blackout in their lifetime and 55.0% reported having experienced a partial blackout in their lifetime. 28.8% reported experiencing a blackout of either type during the past year. These numbers are consistent with the literature reporting that lifetime blackout prevalence lies somewhere between 20 and 55%, and that partial blackouts are more commonly endorsed than are total blackouts (Kypri et al., 2009; Rose and Grant, 2010; Wetherill and Fromme, 2016; White et al., 2002). Our findings add to the literature by providing further context regarding the total (en bloc)/partial (fragmentary) breakdown of lifetime blackout prevalence. Response frequency for select items pertaining to blackout history are displayed in Table 4, and blackout response variables are displayed according to sex/DoA group in Table 5.

**Table 4:** Doa and blackout histories.

Factor	*N*	% of total sample
Total blackout		
Past year incidence	43	12.0
Lifetime incidence	114	31.8
Never experienced	244	68.2
Partial blackout		
Past year incidence	99	27.7
Lifetime incidence	197	55.0
Never experienced	161	45.0
Sleepwalking		
Recent incidence	14	3.9
Lifetime incidence	54	15.1
Never experienced	304	84.9
Sleep terrors		
Recent incidence	34	9.5
Lifetime incidence	75	20.9
Never experienced	283	79.1
Confusional arousals		
Recent incidence	28	7.8
Lifetime incidence	54	15.1
Never experienced	304	84.9

**Table 5:** Blackout histories by sex and DOA group.

Factor	Full sample (*N* = 358)		Males—no DoA (*n* = 121)		Males—DoA (*n* = 47)		Females—no DoA (*n* = 111)		Females—DoA (*n* = 79)	
	*n*	%	*n*	%	*n*	%	*n*	%	*n*	%
background										
Past year incidence	43	12.0	13	10.7	6	12.8	6	5.4	18	22.8
Lifetime incidence	114	31.8	36	29.8	19	40.4	25	22.5	34	43.0
Never experienced	244	68.2	85	70.2	28	59.6	86	77.5	45	57.0
Partial blackout										
Past year incidence	99	27.7	27	22.3	16	34.0	23	20.7	33	41.8
Lifetime incidence	197	55.0	58	47.9	31	66.0	56	50.5	52	65.8
Never experienced	161	45.0	63	52.1	16	34.0	55	49.5	27	34.2

### 3.3 Parasomnia findings

With regard to disorders of arousal, 35.2% of our sample endorsed having ever experienced sleepwalking, sleep terrors, or confusional arousals. Specific breakdowns by episode type are displayed in Table 4.

### 3.4 The relationship between alcohol use, DoA, and ARB susceptibility

#### 3.4.1 Factors predicting alcohol-related blackout in males

**Figure 1:**
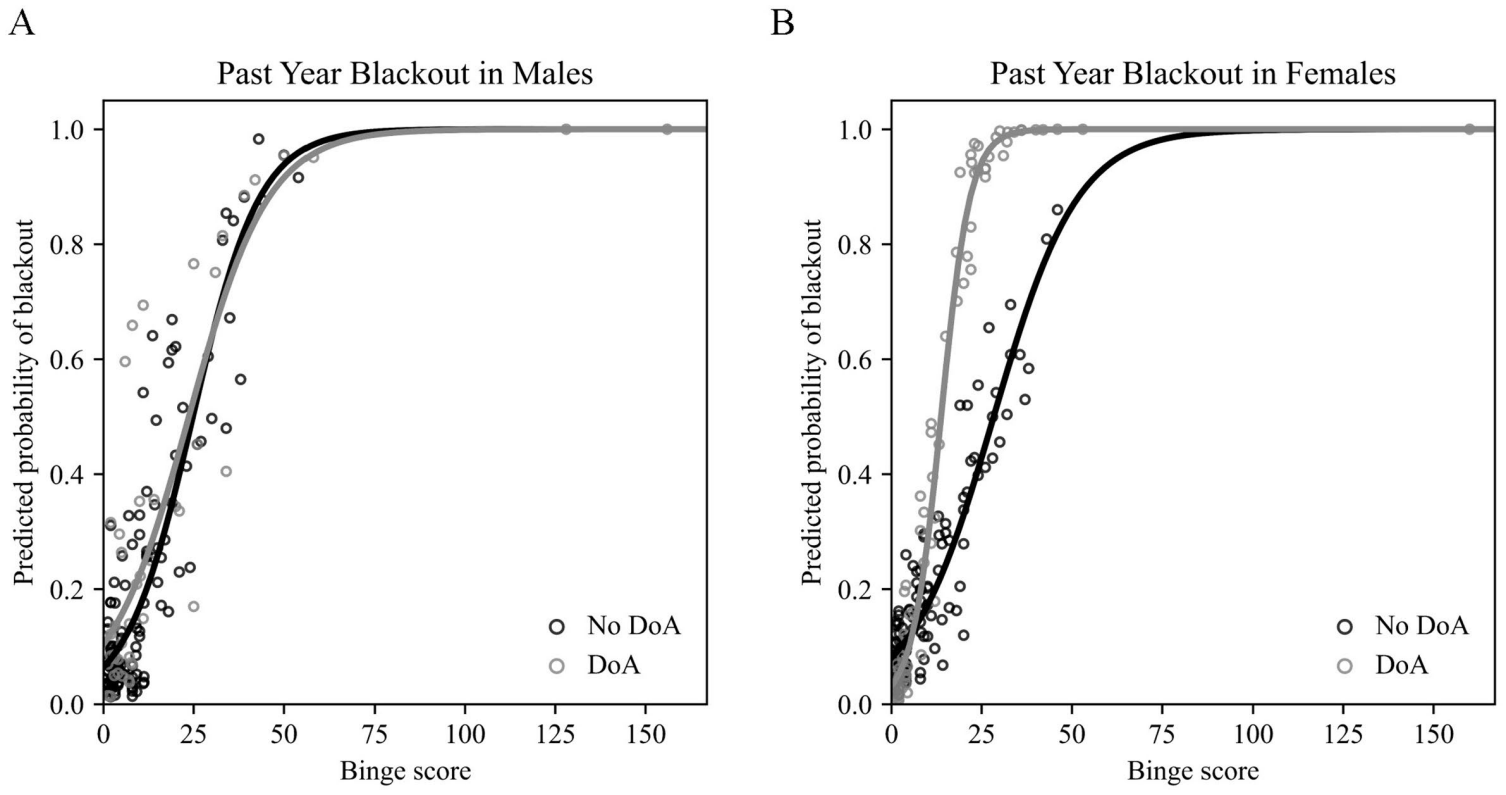
Results of the binary logistic regression models assessing the effects of disorder of arousal (DoA) history and past year binge score in predicting blackout likelihood. (**A**) Past year blackout probabilities predicted by the model for males (*n* = 168) plotted against binge score. No DoA history plotted in black and positive DoA history plotted in gray. (**B)** Past year blackout probabilities predicted by the model for females (*n* = 190) plotted against binge score. No DoA history plotted in black and positive DoA history plotted in gray. DoA, disorders of arousal.^*^Figure made using a custom Python script.

In males, the overall model was significant at a level of *p* < 0.001 (likelihood ratio of full model vs. intercept-only model = 66.223; Nagelkerke R^2^ = 0.477). As expected, we found a significant linear relationship between past year alcohol binge score and past year blackout history (*OR = 1.100*, *p* < 0.001). Greater self-reported psychopathology (*OR = 1.021, p = 0.012*) and higher educational attainment (*OR = 1.424*, *p* = 0.008) were significant predictors of past year blackout probability in this model. Inconsistent with our hypothesis, binary DoA history was not a significant predictor of past year blackout in males (*OR = 0.858*, *p* = 0.855), and did not moderate the effect of binge score on blackout likelihood (interaction *OR = 1.003, p = 0.940*). Family history density, age, race, number of binges before age 18, and substance use were not significant predictors of past year blackout in males in this model (see Table 6). The predicted past year blackout probabilities output by this model are plotted according to binge score in Figure 1A.

**Table 6:** Binary logistic regression model predicting presence or absence of ARB in the past year for males (*n* = 168).

Parameter	Odds ratio	95% confidence interval (LL, UL)	Wald chi-square	Significance (*p*)
Intercept	0.000	–	13.924	< 0.001
Binary DoA history	0.858	0.166, 4.430	0.033	0.855
Binge score	1.100	1.046, 1.158	13.487	<0.001
Number of binges before 18	0.999	0.989, 1.008	0.089	0.765
Total ASR score	1.021	1.005, 1.037	6.299	0.012
Family history density	1.428	0.889, 2.295	2.166	0.141
Age	0.992	0.951, 1.035	0.140	0.708
Race (non-white vs. white)	1.373	0.458, 4.112	0.320	0.572
Substance use score	1.049	0.956, 1.151	1.030	0.310
Education level	1.424	1.099, 1.846	7.131	0.008
Binary DoA history × binge score	1.003	0.921, 1.093	0.006	0.940

OR > 1 indicates increased ARB probability.

In males, individuals reporting any history of DoA had higher ASR scores (*t* = −4.472, *p* < 0.001), greater recent substance use (*t* = −2.634, *p* = 0.010), and younger ages (*t* = 2.597, *p* = 0.011) than those with no DoA history.

#### 3.4.2 Factors predicting alcohol-related blackout in females

In females, the overall model was significant at a level of *p* < 0.001 (likelihood ratio of full model vs. intercept-only model = 96.854; Nagelkerke *R*^2^ = 0.562). Consistent with expectations (and the results in males), we found a significant relationship between past year alcohol binge score and past year blackout (*OR = 1.081, p = 0.002*). Unlike the results of the model run in males, greater psychopathology (*OR = 0.995, p = 0.481*) and higher educational attainment (*OR = 1.050, p = 0.654*) were not significant predictors of past year blackout history in females. Binary DoA history was not in itself a significant predictor of past year blackout in females in the final model (*OR = 0.275, p = 0.149*), but did interact with binge score (*OR = 1.216, p = 0.002*) to predict a steeper increase in likelihood of blackout in those with a history of DoA. In the initial model, which did not include the interaction term, there was a main effect of binary DoA history (*OR = 3.095, p = 0.012*). The main effect of binge score and the binge score by DoA history interaction term were the only significant predictors in the final model; see coefficient values for family history density, age, race, number of binges before age 18, and substance use in Table 7. The predicted past year blackout probabilities output by this model are plotted according to binge score in Figure 1B.

**Table 7:** Binary logistic regression model predicting presence or absence of ARB in the past year for females (*n* = 190).

Parameter	Odds ratio	95% confidence interval (LL, UL)	Wald chi-square	Significance (*p*)
Intercept	0.406	–	0.229	0.602
Binary DoA history	0.275	0.048, 1.586	2.085	0.149
Binge score	1.081	1.030, 1.135	10.032	0.002
Number of binges before 18	1.000	0.991, 1.009	0.006	0.938
Total ASR score	0.995	0.980, 1.009	0.497	0.481
Family history density	1.396	0.857, 2.273	1.796	0.180
Age	0.961	0.921, 1.003	3.385	0.066
Race (non-white vs. white)	1.076	0.402, 2.878	0.021	0.884
Substance use score	0.948	0.854, 1.051	1.035	0.309
Education level	1.050	0.849, 1.297	0.201	0.654
Binary DoA history × binge score	1.216	1.074, 1.377	9.487	0.002

OR > 1 indicates increased ARB probability.

It should be noted that in females, individuals reporting any history of DoA had greater self-reported psychopathology (*t = −6.041, p < 0.001*), higher density of familial problem drinking (*t = −3.551, p < 0.001*), and greater recent substance use (*t = −2.889, p = 0.005*) than those with no DoA history.

## 4 Discussion

### 4.1 The relationship between disorder of arousal symptoms and blackout

The results of this study indicate that DoA history might moderate the relationship between alcohol use and blackout probability in females, such that blackout becomes likely at lower levels of drinking in those who report DoA episodes at any point in their lifetime. It is important to note that when an interaction term between DoA history and binge score was included, DoA history in itself was not a significant predictor of past year blackout. We interpret this as meaning that history of DoA alone cannot predict blackout, but it may strengthen the relationship between alcohol use and blackout, suggesting increased vulnerability to blackout in those with DoA. Interestingly, this relationship was restricted to females in this sample. We would like to avoid framing these results as mechanistically informative, although we will contextualize them with our interpretation with regard to possible underlying neurophysiology. We hypothesize that individuals who experience DoA and individuals who are prone to blackout may have shared instability in intrinsic functional connectivity between the motor cortex and other brain regions (Castelnovo et al., 2016). Such instability may be behaviorally negligible under most circumstances, but manifests as a pathological behavioral state during alcohol intoxication or non-rapid eye movement (NREM) sleep. In individuals with DoA, electroencephalography (EEG) data collected during sleep has revealed patterns of increased beta band activity (13–30 Hz) in the parietal motor and occipital cortices preceding episodes (Castelnovo et al., 2018; Castelnovo et al., 2016; Terzaghi et al., 2009). In terms of slow-wave activity typically associated with sleep, decreases in functional connectivity in the delta band (1–4 Hz) between parietal and occipital channels have been documented prior to somnambulistic (sleepwalking) episodes, despite increases in high frequency connectivity (Desjardins et al., 2017). Opposing shifts in connectivity at lower and higher frequencies may contribute to pathological arousal. Aberrant patterns of waking brain activity have also been documented in sleepwalkers (Oliviero et al., 2007; Rothacher et al., 2020). During the completion of a motor task, sleepwalkers displayed different patterns of beta band activity than did controls (Rothacher et al., 2020). Using paired-pulse transcranial magnetic stimulation (TMS), Oliviero et al. (2007) found evidence of impaired inhibitory control of the motor cortex in sleepwalkers, indicating abnormal GABAergic modulation of the region (Di Lazzaro et al., 2007; Oliviero et al., 2007; Zeugin and Ionta, 2021). Notably, DoA episodes also occur in a minority of people when taking the GABA_A_ receptor positive modulator, zolpidem (Dolder and Nelson, 2008), with greater incidence in women (Joung, 2023) and among carriers of genetic variance in the GABA_A_ receptor that has also been linked to blackout and AUD risk (Dick et al., 2006; Tsai et al., 2013). We thus speculate that functional network instability in both DoA and ARB could reflect atypical network synchronization by GABAergic interneurons (Gonzalez-Burgos and Lewis, 2008; Klausberger and Somogyi, 2008).

Insufficiency in cortical GABAergic circuitry, potentially resulting in failure to inhibit inappropriate activity in the motor cortex during sleep, is thought to be a predisposing factor for DoA (Oliviero et al., 2007). GABA release is important in synchronizing activity both locally and across long-range networks, and abnormal GABAergic modulation of circuitry could be an endophenotype associated with sleepwalking (Kapogiannis et al., 2013; Kiviniemi et al., 2005; Nasrallah et al., 2017). Of course, individual variability in the GABA system is also involved in determining ethanol sensitivity (Davies, 2003). Abnormal GABAergic modulation of circuitry, particularly of circuits involved in encoding episodic memories, is theorized as a contributing factor in blackout susceptibility (Rose and Grant, 2010). It is possible that both behavioral pathologies, disorders of arousal and blackout, share a foundation in aberrant development of GABAergic signaling and subsequent instability of cortical functional connectivity. This hypothesis should be further investigated using methods that can assess cortical excitatory/inhibitory balance.

An alternative, although not mutually exclusive, explanation is that the endophenotypes underlying DoA history may exacerbate group-level differences in sensitivity to the effects of alcohol between males and females. Females tend to have less body water per pound than do males, leading to increased concentrations of alcohol in the blood at similar levels of consumption (Mumenthaler et al., 1999). If any increase in blackout risk is endowed by DoA history, it may be negated by pharmacokinetic factors in males.

### 4.2 Prevalence of DoA in this sample

According to responses on the MUPS, 35.2% of our sample reported any lifetime history of DoA episodes. These data are consistent with existing literature which places the prevalence of a lifetime DoA between 13 and 39% (Loddo et al., 2019). However, 15.9% of our sample endorsed currently experiencing DoA symptoms, which is higher than is typically reported for adults, with prevalence often indicated to be around 2% (Ohayon et al., 1999). This discrepency may be related to our comprehensive assessment of the three DoA episode types, given that most of the previous work assessing prevelance focused exclusively on sleepwalking. Consistent with recent research reporting a prevalence of 3.6% for past-year sleepwalking alone, 3.9% of our sample reported a recent history of sleepwalking (Ohayon et al., 2012). This suggests that this particular parasomnia may be less common than the other DoA episode types. In terms of the other DoA, 9.5% of our sample reported a recent history of sleep terrors and 7.8% reported a recent history of confusional arousals. As sleep terrors and confusional arousals are not as well characterized in the literature, the numbers reported for this sample may provide insight into the specific natures of the different DoA.

### 4.3 Potential mechanisms of sex-differences in the relationship between DoA and ARB susceptibility

Our findings suggesting a possible sex-specific relationship between DoA history and blackout propensity could reflect divergent mechanisms of blackout in males and females. Alternatively, sex differences in neuroactive steroid-mediated GABAergic modulation of connectivity between the motor cortex and other regions could underlie sex differences in blackout. Both progesterone and estradiol are typically present at higher levels in premenopausal females relative to males (Smy and Straseski, 2018; Wirth et al., 2007). Allopregnanolone, a neuroactive steroid derived from progesterone, is an endogenous GABA_A_ receptor modulator that is synthesized in the vertebrate brain (Puia et al., 1990; Reddy, 2010). Additionally, circulating estrogen can modulate synaptic activity via receptor binding in the brain, including at inhibitory interneurons in the hippocampus (McEwen et al., 2001; Nilsson et al., 2001). It is possible that hormonal fluctuations in neuroactive steroids influence GABAergic modulation of functional connectivity in female individuals (Greenwell et al., 2023), and in some individuals contribute to episodes of disturbed regional synchronization with behavioral ramifications. Previous research has demonstrated significant cognitive impairment in women during acute alcohol intoxication compared to men, but little difference between men and women in motor function under the influence of alcohol (Rose and Grant, 2010). This may indicate that alcohol exerts general, brain-wide impairments in men while having more localized effects on women, which may be related to steroid-mediated GABAergic modulation of functional connectivity between networks. The mechanisms proposed here are entirely speculative, and beyond the scope of this project. These complex processes will need to be further explored in future research.

### 4.4 Limitations of the current study

We acknowledge several limitations of the present study. The first is the skewed distribution of the variables of interest, including the parasomnia variables, consistent with the fact that DoA are relatively uncommon in the general population (Stallman and Kohler, 2016). Future studies that examine the connection between DoA and ARB could instead specifically recruit individuals with disorder of arousal history using a clinical recruitment database. Another potential limitation is our use of a binary blackout variable collapsing across past year partial/fragmentary and total/en bloc blackouts as our dependent variable. The two types of ARB are behaviorally distinct in the degree of amnesia experienced, but they may or may not be mechanistically distinct. Because blackouts of either type were generally reported with a low frequency in this sample, we combined the two to increase power. Future studies with samples specifically recruited for high frequency of ARB episodes are needed to determine whether partial and total blackouts represent different processes or potentially lie on a spectrum of dysconnectivity. Additionally, it should be noted that the ASR, which we used to quantify psychopathology, has only previously been validated up to age 59 (Ivanova et al., 2015). The maximum age in this sample was 72 years old, which should be noted in interpreting this score for the 26 participants between 60 and 72 years old included in these analyses. Additionally, the use of incongruent timelines for our variables of interest may be considered a limitation, as our model included measures of drinking behavior and blackouts during the past year, with DoA history assessed across the lifetime. These variables were chosen to control for periods of drinking history not assessed by our alcohol use measures. The AUDIT captures drinking during the past year, and the CAUPQ assesses drinking during the past year, before age 18, and between ages 18 and 21. Because there is a very clear relationship between alcohol consumption and ARB, we wanted to tightly control the time periods included (Labrie et al., 2011; Wetherill and Fromme, 2016). Using drinking and ARB measures from the past year was also intended to minimize any confounds related to recall and age. We used lifetime DoA history because our hypothesis was not that recent DoA episodes trigger blackouts as binge drinking does, but rather that DoA and ARB may share underlying pathophysiology. As the accuracy of recall for DoA episodes early in the lifetime may wane with age, we used age as a covariate in all models. The use of these variables also provides some clarity on the directionality of these effects, as it indicates that any relationship between ARB and DoA cannot be the result of only sleep disruption due to alcohol use. As mentioned previously, in both males and females, the DoA groups differed statistically on multiple relevant variables. Most notably, females with a history of DoA reported significantly higher substance use scores than those without a history of DoA. They also reported higher binge scores, although this difference did not meet statistical significance. It is entirely possible that these group differences contributed to our findings, and that escalation in drinking and substance use in the DoA group account for differences in blackout likelihood. On the other hand, if this were the case it seems unlikely that the effects would manifest as an interaction. Furthermore, it would not explain the sex-dependent interaction effect, as the same group differences exist in the males in this sample. Finally, self-report data collection comes with inherent questions of reliability, particularly for measures related to substance use. However, given the experimentally-challenging nature of ARB research, self-report data collection remains one of the only suitable investigational options. Moreover, the nature of low-cost online self-report data collection provides the advantage of including a wider range of individuals than those typically included in in-person experiments.

### 4.5 Conclusion

The purpose of this study was primarily to establish whether DoA history was related to ARB history in a sample of adults, even when controlling for alcohol use. These findings suggest a sex-dependent link between the two states, such that history of DoA episodes may share underlying phenotypes with susceptibility to blackout in females. Experiencing ARBs can be detrimental to one’s health, safety, social life, and academic or career pursuits. The findings presented here represent a novel approach to understanding ARB, and help shed light on potential predisposing factors. Future work is needed to explore this connection, particularly sex differences in the mechanisms underlying both ARB and DoA. We anticipate that individuals who are prone to blackout may demonstrate atypical patterns of functional connectivity between the motor cortex and prefrontal cortex or hippocampus while sober and at-rest—a hypothesis that could be examined using neuroimaging methods such as resting-state EEG or fMRI. The findings described here provide direction for future work on ARB, which may aid in identifying those most at risk for AUD development and other alcohol-related harms.

## Data Availability

The raw data supporting the conclusions of this article will be made available by the authors, without undue reservation.

## Footnotes

1
https://www.prolific.co
