# Supplementary material for: Sex differences in blackout: evidence of a relationship between disorders of arousal during sleep and alcohol-related blackout in females
Source: Front Psychol. 2026 Feb 12;17:1703614. doi: 10.3389/fpsyg.2026.1703614 (PMC12936044; doi:10.3389/fpsyg.2026.1703614)

**Table S1:** Demographic variables by sex and lifetime blackout groups

|  | **Full Sample**  **(N = 358)** | | **Males – No AIB**  **(n = 75)** | | **Males – AIB**  **(n = 93)** | | **Females – No AIB**  **(n = 76)** | | **Females – AIB**  **(n = 114)** | |
| --- | --- | --- | --- | --- | --- | --- | --- | --- | --- | --- |
| ***Race*** | **n** | **%** | **n** | **%** | **n** | **%** | **n** | **%** | **n** | **%** |
| White | 264 | 73.7 | 49 | 65.3 | 75 | 80.6 | 50 | 65.8 | 90 | 78.9 |
| Non-White | 94 | 26.3 | 26 | 34.7 | 18 | 19.4 | 26 | 34.2 | 24 | 21.1 |
| ***Ethnicity*** | **n** | **%** | **n** | **%** | **n** | **%** | **n** | **%** | **n** | **%** |
| Hispanic | 75 | 20.9 | 14 | 18.7 | 21 | 22.6 | 13 | 17.1 | 27 | 23.7 |
| Non-Hispanic | 283 | 79.1 | 61 | 81.3 | 72 | 77.4 | 63 | 82.9 | 87 | 76.3 |
| ***Education Level*** | **n** | **%** | **n** | **%** | **n** | **%** | **n** | **%** | **n** | **%** |
| Bachelor’s Degree or higher | 208 | 58.1 | 39 | 52.0 | 66 | 71.0 | 37 | 48.7 | 66 | 57.9 |

**Table S2:** Correlation matrix for continuous variables included in the model (Spearman’s rho)

|  | Number of blackouts past year | Binge score | Number of binges before age 18 | Total ASR score | Family history density | Age | Substance use score | Education level |
| --- | --- | --- | --- | --- | --- | --- | --- | --- |
| Number of blackouts past year | 1.000 |  |  |  |  |  |  |  |
| Binge score | 0.583*** | 1.000 |  |  |  |  |  |  |
| Number of binges before age 18 | 0.114* | 0.183*** | 1.000 |  |  |  |  |  |
| Total ASR score | 0.294*** | 0.269*** | 0.009 | 1.000 |  |  |  |  |
| Family history density | 0.225*** | 0.223*** | 0.142** | 0.257*** | 1.000 |  |  |  |
| Age | -0.242*** | -0.360*** | 0.030 | -0.360*** | -0.114* | 1.000 |  |  |
| Substance use score | 0.261*** | 0.325*** | 0.219*** | 0.235*** | 0.201*** | -0.112* | 1.000 |  |
| Education level | -0.029 | -0.099 | -0.057 | -0.245*** | -0.137** | 0.209*** | -0.233*** | 1.000 |
| DoA severity | 0.219*** | 0.102 | -0.030 | 0.444*** | 0.246*** | -0.124* | 0.251*** | -0.107* |

**Table S3:** Binary logistic regression model predicting presence or absence of ARB in the past year for females (n = 190). OR > 1 indicates increased ARB probability.

| **Parameter** | **Odds ratio** | **95% confidence interval (LL, UL)** | **Wald chi-square** | **Significance (p)** |
| --- | --- | --- | --- | --- |
| Intercept | 0.328 | - | 1.882 | .170 |
| Binary DoA history | 0.312 | 0.059, 1.660 | 1.865 | .172 |
| **Binge score** | **1.075** | **1.027, 1.125** | **9.560** | **.002** |
| Age | 0.966 | 0.930, 1.004 | 3.116 | .078 |
| **Binary DoA history * Binge score** | **1.194** | **1.065, 1.339** | **9.288** | **.002** |

**Table S4:** Binary logistic regression model predicting presence or absence of ARB in the past year for males (n = 168). OR > 1 indicates increased ARB probability.

| **Parameter** | **Odds ratio** | **95% confidence interval (LL, UL)** | **Wald chi-square** | **Significance (p)** |
| --- | --- | --- | --- | --- |
| Intercept | 0.098 | - | 6.841 | .009 |
| Binary DoA history | 1.612 | 0.383, 6.778 | 0.424 | .515 |
| **Binge score** | **1.111** | **1.059, 1.165** | **18.425** | **< .001** |
| Age | 0.992 | 0.956, 1.029 | 0.195 | .658 |
| Binary DoA history * Binge score | **0.983** | **0.911, 1.061** | **0.191** | **.662** |


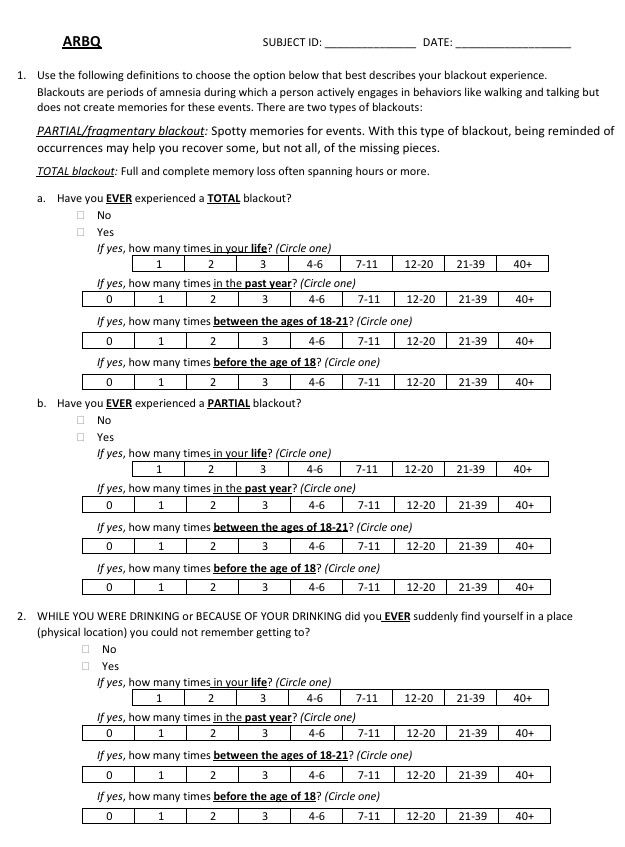


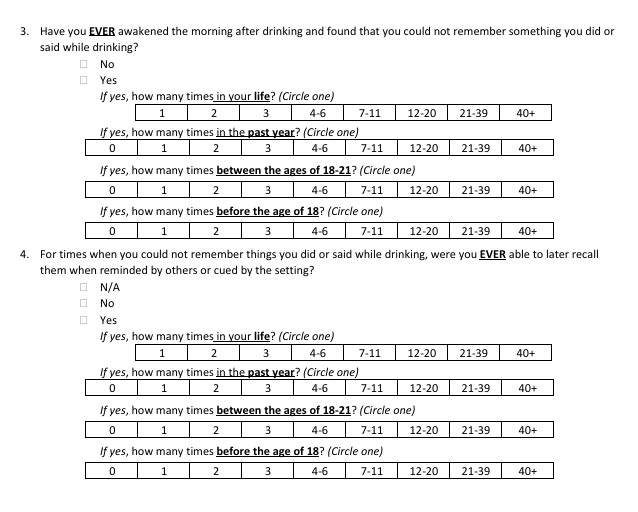

Supplement: Supplementary file 1 [file Table_1.DOCX]
